# Teacher and Student Experiences during First-Trial Chemistry Lessons in Immersive Virtual Reality

**DOI:** 10.1021/acs.jchemed.6c01221

**Published:** 2026-10-12

**Authors:** Mária Babinčáková, Paweł Bernard

**Affiliations:** 201871 Jagiellonian University in Kraków, Faculty of Chemistry, Department of Chemical Education, ul. Gronostajowa 2, 30-387 Kraków, Poland

**Keywords:** High School, Chemoinformatics, Immersive Virtual Reality (IVR), Multimedia-Based Learning, Molecular Properties/Structure, Stereochemistry, Theoretical Chemistry

## Abstract

Virtual reality (VR) is becoming increasingly popular and is being implemented across many areas and levels of chemistry education. When planning to incorporate this technology into teaching, several factors must be considered, such as the cost of these devices, the time required to prepare a VR lesson, and the exhaustion they can cause. Is it worth it? A 90 min lesson in Nanome was prepared and tested with 32 students from lower secondary schools (K8) and 59 students from upper secondary schools (K11). Feedback was collected through a short questionnaire completed by students and semistructured interviews conducted with teachers. This paper presents experiences, opinions, and evaluations from 91 students and two teachers regarding their first attempt to implement VR-based teaching and learning at the secondary school level. VR offers exciting potential for school chemistry education by overcoming physical laboratory limitations and enabling interactive, immersive molecular modeling that boosts student motivation. However, successful integration requires dedicated technical and spatial support to help teachers navigate hardware challenges, lesson preparation, and varying student reactions based on students’ ages and prior VR experience. Successful VR integration requires clear learning goals and an initial time investment, but once teachers master the technology, reusable lesson plans make the effort well worth it.

## Graphical abstract

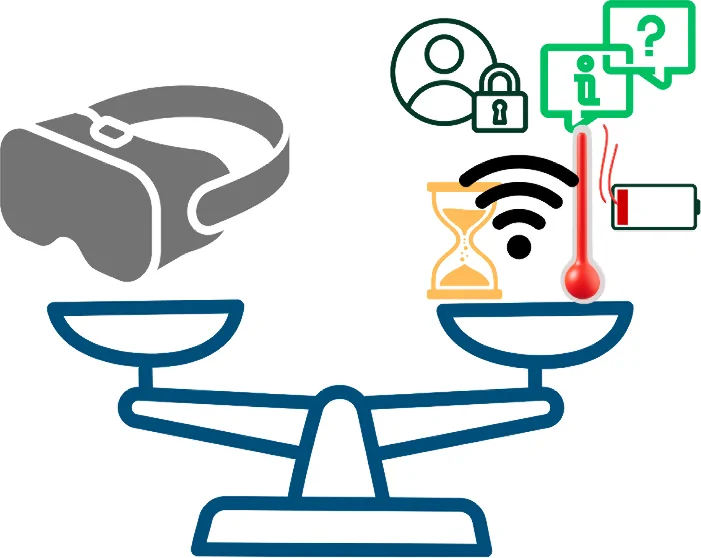

## Introduction

In 2015, Klaus Schwab, former executive chairman of the World Economic Forum (WEF), introduced the Fourth Industrial Revolution (IR 4.0) to a wider audience.^1^ IR 4.0 necessitated change across many sectors, including education. Since then, augmented reality and virtual reality have become very popular in education, even though they originated much earlier.^2^

### Virtual Reality and Immersive Virtual Reality

Initially, virtual reality took the form of interactive, immersive computer simulations.^3^ Later, five key elements of a virtual reality experience were introduced by Sherman and Craig: the virtual world, immersion (physical and mental), interactivity, and the people on the creating and receiving sides of the medium.^4^ This idea developed alongside technological advances.

Virtual Reality (VR) is a computer-generated simulation of a three-dimensional world. Users can interact with this world using specialized electronic equipment, primarily a head-mounted display (HMD) and controllers. The goal of VR is to create an illusion that replaces the real world with a digital one, but the user often remains acutely aware of the technology mediating the experience.^5^

Immersive Virtual Reality is the evolution of this concept. It does not just aim to show you a new world; it makes you believe you are in it and forget the old one entirely. The key differentiator is presence—the feeling of actually being in the virtual space.^4^

### Emergence of Virtual Reality Technology

In 1892, the American zoologist Horatio Saltonstall Greenough proposed the first stereomicroscope, which enabled three-dimensional visualization of samples.^6^

Almost 70 years later, in 1962, Morton Heilig introduced the Sensorama, a mechanical device with supporting tools that simulated a motorcycle ride through New York by having the spectator sit on an imaginary motorcycle and experience the street through the screen, fan-generated wind, and simulated city noise and smells.^7^ In 1968, “Ivan Sutherland’s head-mounted 3D display” (HMD) was introduced.^8^ It is considered the first virtual reality stereoscopic head-mounted viewing apparatus. Although it was still quite heavy, it must have been suspended from the ceiling.^7,9,10^

In 1987, Jaron Lanier officially popularized the term ″Virtual Reality.″ His company was the first to sell VR goggles and wired gloves.^11^

Later, in 2006, Nintendo introduced its fifth major game console, the Wii,^12^ which supported wireless connectivity for select games and included sensors that allowed the Wii Remote to recognize its orientation after being moved from a resting position, translating that motion into gesture recognition for a game.^12^

In 2012, the Oculus Rift launched a modern, lightweight, high-resolution VR headset.^13^ The idea became very popular and promising, and in 2014, Facebook (now Meta) bought the company.

As we can see, the modern concept of VR, including immersive systems, has become more accessible to the public due to advances in hardware/software, multiple forms of government support, and, more importantly, reduced costs.^14,15^

### Implementation of VR in Education

The high cost of the technology initially limited VR’s use to the industry and the military for a wide variety of purposes.^16^ In the early 90s, the first examples of VR in education were published.^17^ Later in the 2000s, the work on implementing VR in education continued.^18^ Around the same time, CAVE systems (cave automatic virtual environment), or rooms in which images are projected onto the walls via displays or projectors, were used in education and exhibitions.^19^

In 2015, Google launched Google Cardboard and Google Expeditions. By turning everyday smartphones into cheap VR viewers, millions of students could suddenly take virtual field trips, marking the first widespread adoption of VR in primary and secondary education.^20^

Lately, VR technology has become even more popular and available in schools, especially after the pandemic.^21^

### Implementation of VR in Chemistry Education

Given the nature of chemistry, VR offers several opportunities in the educational process. Here, the most relevant and well-known VR solutions/platforms for goggles are described (Table 1), including laboratories, simulations, game-based learning, the periodic table of elements, research environment tools, and educational environments. The examples were selected because they are recent, recognized, and widely used in teaching practices and published journals.

**Table 1:** Examples of How to Implement VR in Chemistry Education, with Specific Solutions

parameter	comment
**Virtual laboratories**	ChemersiveLLM – laboratory (ChemersiveLLM that integrates Large Language Models with Virtual Reality to enhance chemistry education through immersive, interactive, and intelligent virtual lab experiences^22^
	Vic’s Science Studio – Chemistry lab software for VR headset, ready-to-use task scenes^23^
	Hands-On Virtual-Reality (HOVR) Lab^24^
	ChemistryVR – observation of chemical reactions, building molecular models, etc.^25^
**Simulations**	iMD-VR – molecular dynamics simulations^26,27^
	NOMAD VR – a simulation platform^28^
**Game-based learning**	Virtual Chemistry Classroom for Chemical Bonding - Virtual Chemistry Classroom for Chemical Bonding (VC3B) to facilitate the learning of chemical bonding and formulas through a game-based learning approach, with molecules only in 2D^29^
	VR Stereo Thrills & Spills – an immersive virtual reality game for enhancing student understanding of stereoisomerism^30^
	The Masked Scientist – a chemical escape room about the model of the atom, radioactivity, and the periodic table.^31^
**Periodic table**	MolecularWebXR.org – an augmented or virtual reality tool for the periodic table^32^
**Research Environment tools**	Nanome – primarily aimed at collaborative drug discovery^33^
	VisionMol – a tool for protein molecular structure visualization and manipulation^34^
**Educational Environment**	VeRidium – an interactive virtual reality environment for the utilization in university-level teaching, available since 2025 ^35^

It is important to mention that VR systems are rapidly evolving. What works one day may not be available the next day.

### Learning Outcomes from Implementing Virtual Reality in Secondary Chemistry Education

There are several documented learning outcomes from implementing VR in secondary chemistry education. They include enhanced conceptual understanding of complex chemical topics (such as molecular structures, bonding, and reaction mechanisms), improved spatial reasoning skills, and increased student engagement and motivation.^36–39^ These studies suggest that further research about VR at the secondary school level is missing.

### Spatial Literacy

One of the many advantages VR offers is the opportunity to develop spatial literacy. As Johnstone introduced in the early 80s, the difficulty of chemistry as a subject may be caused by the representation of chemical substances at three levels: macroscopic (properties of matter that can be determined organoleptically), (sub)­microscopic (interactions between entities), and symbolic (chemical formulas).^40,41^ Students have to master the description of matter at all three levels and be able to conclude on macroscopic properties of matter from its structure. It requires a high level of imaginative involvement from a person (student).

VR shows potential in developing spatial literacy in chemistry education.^42,43^ This is one of the main reasons why the topic of spatial literacy and VR was chosen.

### Are We ready?

Below, an article focused on the personal experiences of tutors and students is presented. Its aim is to answer the following questions:

- Are currently available VR systems user-friendly enough to be used in everyday school practice?
- Is this technology ready for implementation in the chemistry classroom at secondary school level?

As mentioned before, only a few examples of VR implementation in secondary-level chemistry education have been described, and they do not focus on practical aspects and existing challenges.

## Methodology

The initial plan was to prepare a 90 min lesson in virtual reality (VR) focused on the structure of molecules and the transition between formula-structure-properties of substances. The first step was purchasing VR goggles—one set for the teacher and five sets for the students. At the beginning of the research, the release of the new Oculus Quest had been anticipated, promising advanced multifunctional sensors. However, the eye-tracking-enhanced sensors were only available in the Pro version; the price (over $1,000 per set) exceeded the budget. Consequently, six sets of the Oculus Quest 3 (cost $550 per set in spring 2024) were purchased. Unfortunately, the newly — at that time released software for group management of the goggles also exceeded our budget.

### Preparing the Learning Environment

First, the goggles must have been prepared for use, which was quite a challenging task. Each set of goggles required a separate Meta account, but linking them to personal social media was risky; therefore, dedicated accounts had to be set up. As mentioned before, purchasing a management environment for educational purposes was not possible. Once everything was set up, all updates were completed, and each set was ready, attention turned to chemistry-related topics and selecting the appropriate software for the lesson. It is important to mention that, when the lessons were prepared (spring 2024), the Meta account (in Poland) was connected to your Facebook account. Later, the opportunity to create a separate Meta account was added with a developer account option.

The lesson’s main focus was spatial literacy, which limited the options. After careful consideration, Nanome was chosen, a virtual reality software platform designed for molecular modeling, collaborative drug design, and 3D visualization of molecular structures. It was also important that it allows users to create a virtual room where several users can join for discussions and manipulate virtual molecules together. The free version offers limited functionality, especially for team management and group organization in the virtual room. However, following our email correspondence, the company offered us a 2-month Pro version for research and educational purposes (the pricing for academic use is 20.83$ per month – price from September 2026). This Pro version enables creating a secure, password-protected private room, developing and saving lesson scenarios, preparing 3D models of molecules, allowing students to interact with the structures as needed, and, most importantly, providing management tools for the tutor to control the process. The Nanome lesson was crafted and it was very time-consuming, taking over 30 h with VR goggles to prepare to a satisfactory standard.

### Lesson Scenarios

The authors prepared the lesson scenario and lecture. Two versions of the lesson were prepared: one for the K8 level and one for the K11 level. The didactic goals of the lessons were:

- Practicing the ability to relate the structure of matter to its properties. (How does the structure of a molecule—polar or nonpolar—affect the physical properties of a substance?) (K8 & K11)
- Review the concept of chemical bond polarity. Familiarize students with the method for determining the polarity of chemical molecules. (K8 & K11)
- Familiarize students with writing structural formulas that take into account the spatial structure of a molecule (bonds in front of and behind the ″plane of the paper″). (K8 & K11)
- Familiarize students with the concepts of optical isomerism, chirality, enantiomers, and the absolute configuration of R and S isomers. (K11)
- Familiarize students with structural formulas that take into account the spatial structure of a molecule (bonds in front of and behind the ″plane of the page″) will be developed. (K11)
- Develop the ability to manipulate models of chemical molecules mentally. (K11)

The topic (and didactic goals) was chosen so that students would have minimal prior knowledge of the study materials and a similar starting point for learning. The teacher’s lesson scenario is in Supporting Information 4: Lesson scenario for teacher.

### Participants

Students worked in small groups (up to 6) due to the number of goggles. The first (pilot) study was done in June 2024. Here, 11 students from 3 schools participated. This session aimed to observe how students interact with the environment and identify the main difficulties.^39^

The main study took place in spring 2025 at the Jagiellonian University in Kraków, Faculty of Chemistry, and involved both lower- and upper-secondary students. The study included 59 students from 12 upper secondary schools (K11) and 32 students from 8 lower secondary schools (K8), with consent from their parents or legal guardians. The selected schools regularly cooperate with the university. Students participated voluntarily. It was an extracurricular activity, but it was related to the chemistry school curriculum. Each student used a separate VR set and worked individually in the VR environment under the teacher’s supervision.

Two teachers were teaching in the VR environment to provide a broader view of the method. The first one was one of the authors. The second was a colleague from the institution with no prior experience in VR. Both teachers had technical support provided during the lessons.

### Questionnaire

After the session, students filled out a short, 5-point Likert scale questionnaire (1 – I definitely do not agree, 2 – I rather do not agree, 3 – It is hard to say, 4 – I rather agree, 5 – I definitely agree) to express their attitude toward learning in VR. The final questions and questionnaire were developed by combining selected items from two validated questionnaires, which were then modified to suit the specific context of this study.^44,45^ Questions are available in the Supporting Information 1.

Students’ answers were transcribed and entered into an Excel spreadsheet, where all data were coded. Similarly, basic statistics, such as counts, means, and averages, were calculated here.

### Safety Statement

Some users may experience motion sickness, dizziness, nausea, eye strain, or disorientation while using VR goggles.

## Results

After having the lessons in VR with over 15 groups, several key problematic areas emerged. Here we describe them in more detail. Additionally, we present the perspectives of the students and teachers.

### Account Difficulties

The first step was to log into the goggles. Since we did not use any management system, we handled everything ourselves. Each device needed a new account; otherwise, it could not be used. At the same time, Meta links all accounts. So, if you use your personal account, your Messenger, Instagram, and Facebook accounts will also appear there, and you need to be careful about where they are and whether your students have access to them.

### Equipment Support

The first thing we did after buying the goggles was to name all the sets—headsets and controllers—and write these names on each device. This was necessary because students sometimes mixed them up. Since we had prior experience using VR goggles for gaming, we knew wearing them for longer periods could be uncomfortable. Therefore, each set was equipped with an extra head-holding strap. Additionally, because young students also attended lessons and have smaller heads, these straps were essential for them. We also purchased new cases for the goggles to make storing and transporting easier.

### Internet

We knew it would be challenging to connect six VR goggles to a single, stable network. Therefore, we needed a modern router that provided a fast and stable internal network and was connected to high-speed Internet. We advise using a dedicated router for those purposes and do not allow other devices to access that network. Our router parameters were: maximum wireless transmission speed: 5400 Mb/s (Wi-Fi); connectors: RJ-45 100/1000/2500 (LAN/WAN); operating frequency: 5 GHz.

### Battery

As with all electronic devices, battery life is crucial. Since the producer promised 2–3 h, depending on the app or game, we initially thought it would not be a problem. As we experienced, it lasted about 1.5 h (sometimes less), and the devices started showing a battery warning around 1 h and 15 min. Therefore, we bought power banks as backups. At the same time, we hesitated to connect them to the goggles immediately (only if necessary) because using a cable-connected power bank was problematic and less comfortable, especially when moving the controller. Additionally, it would cause the goggles to overheat. We did not want to add another permanent battery to the goggles to avoid increasing their weight. The setup is visible in Figure 1.

**Figure 1:**
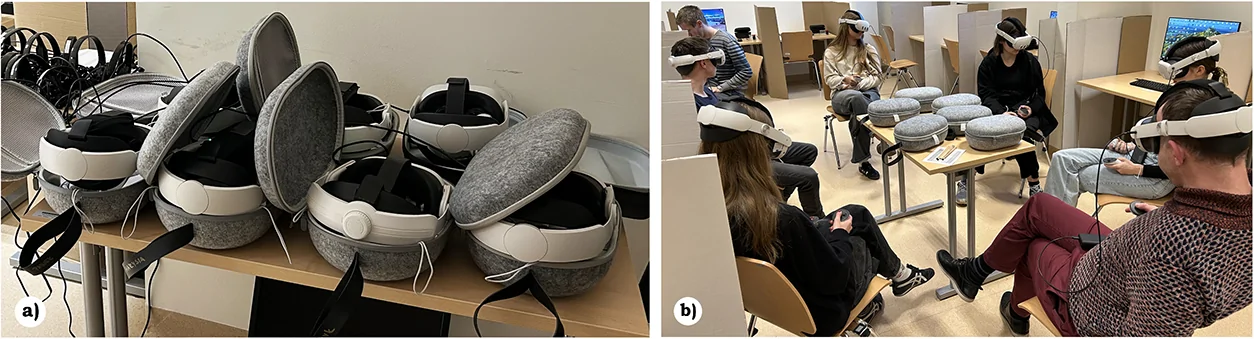
Organizing the VR setup in class: a) Charging station for goggles; b) Students’ arrangement for using VR during the lesson, and charging goggles if necessary.

### Overheating of the Devices

Working in the Nanome environment caused the devices to overheat. Some students reported that the goggles felt very hot and that their heads were getting hot too; other students reported that their goggles were warm. The main issue was the discomfort it caused. This issue was especially noticeable while goggles were powered by power banks.

### Personal/Technical Support

This was a key challenge and solution when working with VR. Since the teacher wants to focus on teaching rather than solving students’ technical issues, having someone available to assist with them is crucial. This person also needs to know the environment very well to guide students without needing to put on their goggles or change their VR boundaries. As we mentioned before, the second author provided the support.

### Preparing the Physical Space

The entire study took place at university building in a standard computer classroom. Students sat on chairs rather than standing, as movement was unnecessary and sitting was easier. Initially, we used wheeled office chairs. However, by the end of the lesson, students had spread out across the room, which created safety concerns. After several sessions, we agreed to seat them in stable chairs that would not be moved around. Additionally, when students were instructed on how to use the goggles, they needed a place to put the boxes, so a table in the center was added. The initial (Figure 2a) and final (Figure 2b) room arrangement are visible in Figure 2.

**Figure 2:**
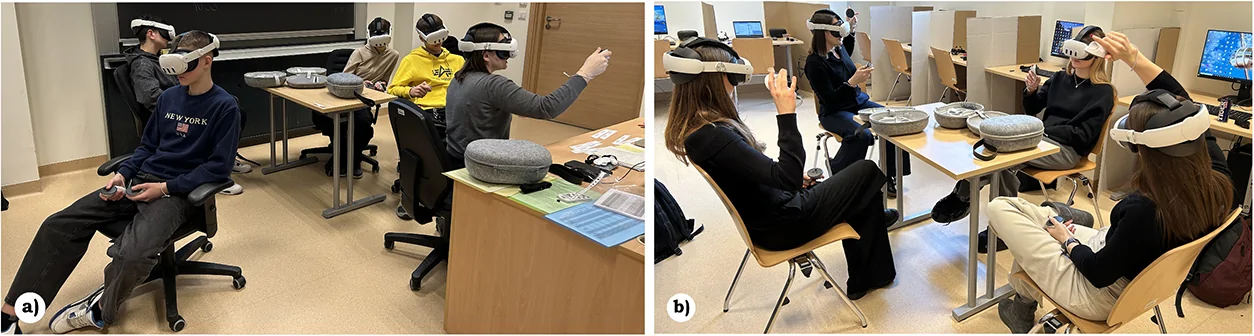
Room’s arrangement during the lesson: a) First arrangement with wheeled chairs; b) Final arrangement with traditional chairs and a table in the middle.

### Teaching Materials

Teacher supporting materials (such as lesson scenarios or properties of chemical substances; see Supporting Information 4: Lesson scenario for teacher) were something we did not initially consider. But as we developed the 90 min scenario, we realized the teacher needed supporting materials to make it easier to follow the prepared lesson. It is not feasible to have “files” or “folders” with documents. The easiest solution was to do it by loading it on our department’s web page in the form of a PDF file, where we placed all necessary materials (Figure 3).

**Figure 3:**
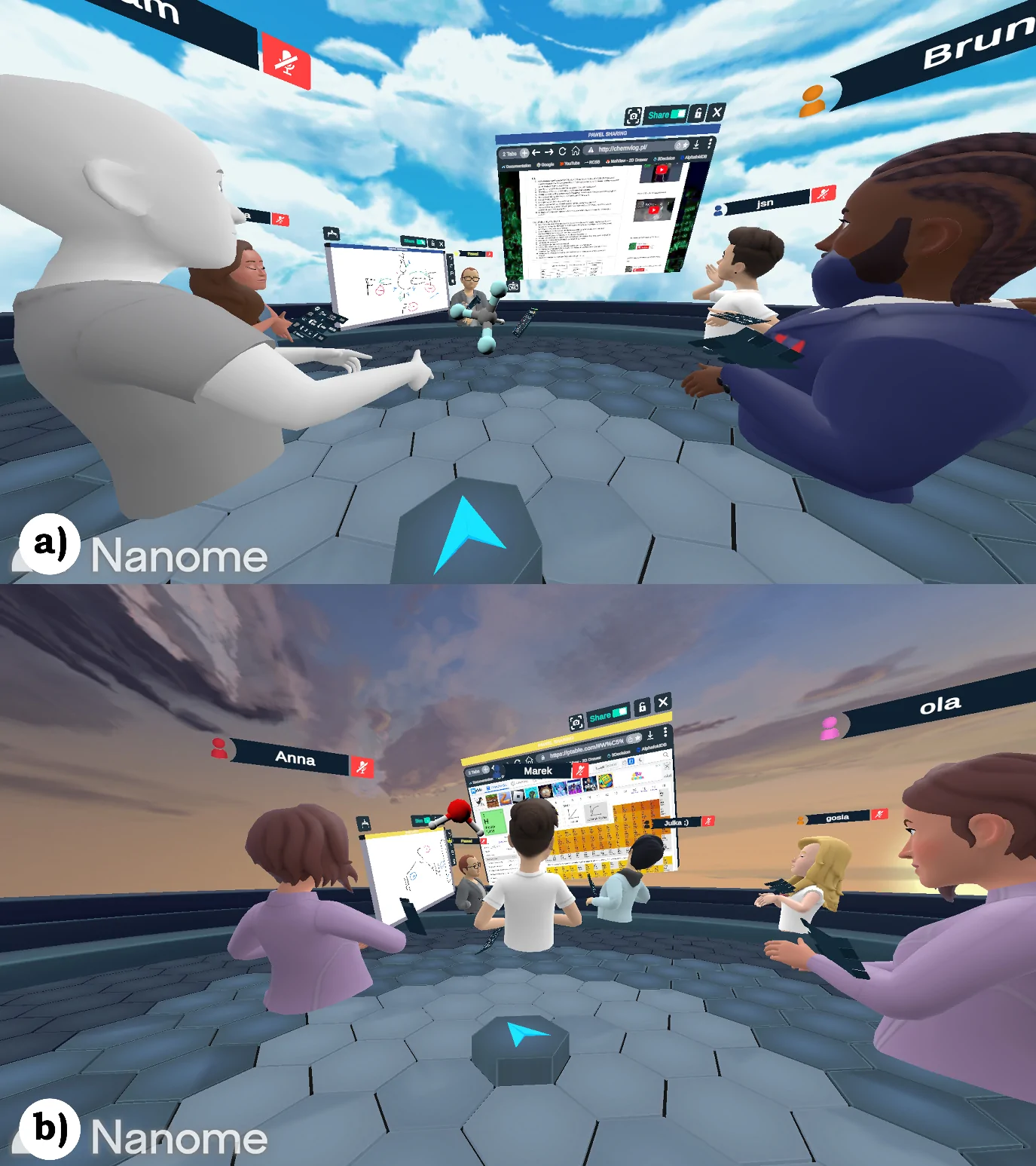
Showing in VR: a) Learning materials and instructions to students; b) The periodic table of elements

### Drawing in Air

Since the lesson was about spatial literacy, drawing molecular structures on a virtual whiteboard was part of the lesson. This appeared to be a tricky issue. In the Nanome, there is an option for every user to turn on a personal virtual whiteboard, write on it, show it, and make it visible for others. It is possible to place this whiteboard horizontally, like a piece of paper, and “draw on a desk”. However, when we tried it with some students, they found it difficult to turn on, which made it very time-consuming. Therefore, students just needed to get used to drawing in the air – drawing on a virtual whiteboard (Figure 4).

**Figure 4:**
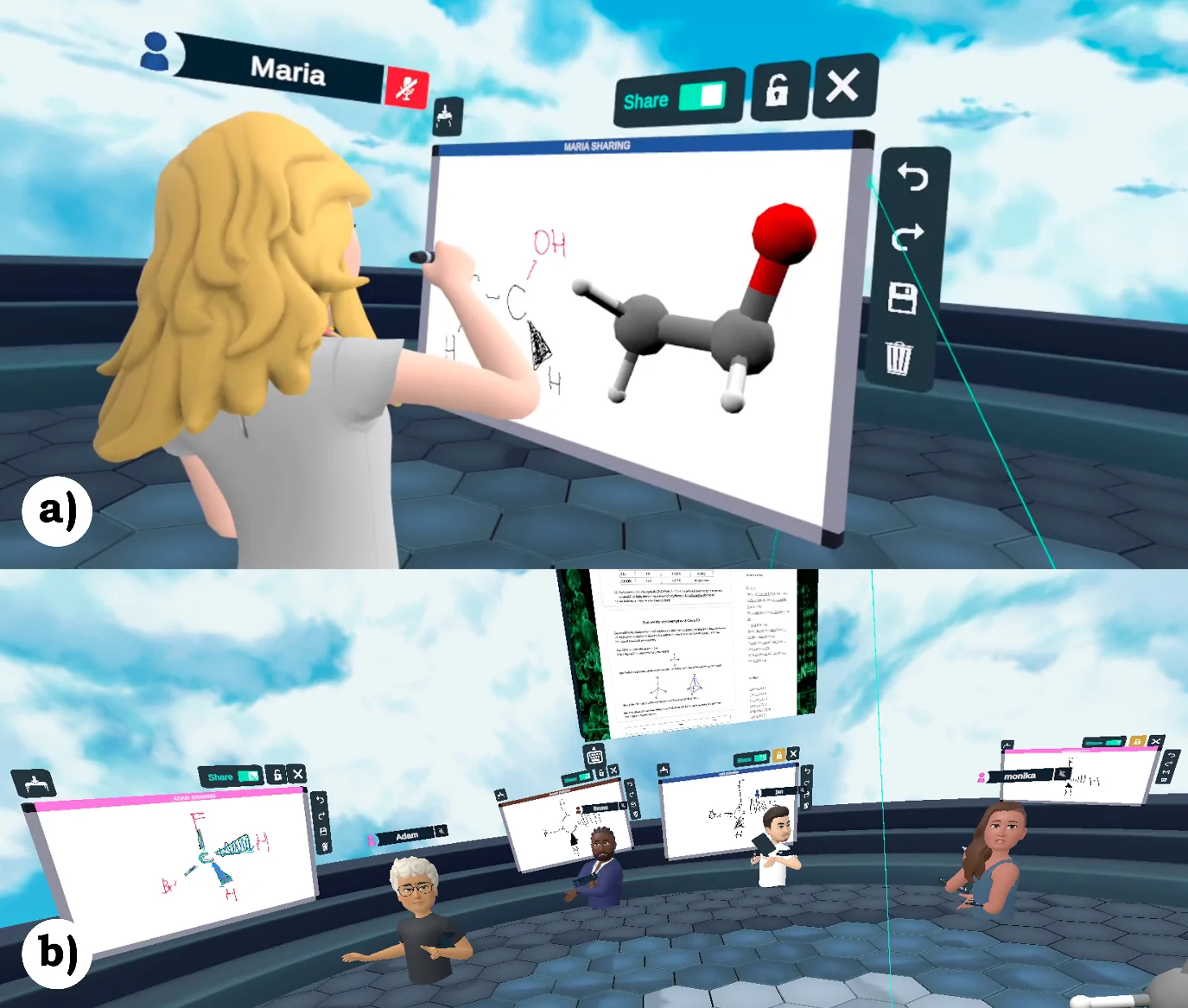
Examples of students’ drawings on the whiteboard – drawing in the air: a) An individual student trying to draw the molecule of ethanol; b) Various examples of drawings, some were nice, some not that much.

### Virtual Reality Sickness

Virtual reality sickness was one thing we were very concerned about before the research. However, during the lessons, we had limited experience with these situations. Usually, it was associated with the heat generated by overheating the devices. There were only 2 cases when students felt a little bit uncomfortable. When students reported this, the Personal/Technical Support person immediately helped them, either by calling their nearby teacher, walking with them for a moment, or by taking them to a place with fresher air.

### Insights from the Teachers’ Point of View

In this section, we present the teachers’ perspectives on teaching in VR based on semistructured interviews. The first experience is from Paweł – one of the authors and cocreators of the lesson scenario. He had previous experience in VR. The second experience is from Ana, who had a limited previous experience with VR and prepared specifically for this lesson. Their detailed perspective is presented in the Supporting Information 2.

#### Previous VR Experience

Paweł had significant prior VR experience (about six months of recreational use before teaching in VR). Ana had no prior VR experience, either educational or recreational. Her first try was for these purposes, and she started with a short game.

#### Initial Adaptation to VR

Paweł focused heavily on the challenge of onboarding students, many of whom were using VR for the first time and experienced a mix of excitement and anxiety. Ana described her own adaptation process, including dizziness and balance problems, especially in games requiring movement. After that, she took off the goggles, and she tried again the next day. But the following times she entered VR, she experienced no problems.

#### Preparation for Teaching

Paweł emphasized preparing teaching materials (PDFs, Web sites, the periodic table, and a molecular repository) and adapting his existing teaching style to VR. Ana spent approximately 4 h preparing, mostly refining the pedagogical concept rather than learning the technology itself. She worked iteratively, testing ideas in short VR sessions.

#### Teaching Approach

Paweł recreated a traditional classroom structure, using presentations, a virtual board, and student-shared boards for note-taking and exercises. Ana focused more on using VR as a visualization tool and preferred experiences where VR felt like an extension of the real environment rather than an immersive virtual world.

#### Student Management

Paweł found student management in Nanome intuitive once everyone had entered the application, and highlighted its ability to move between students and provide immediate feedback. Ana reported that some students became distracted by menus and unrelated VR features, making classroom management more challenging.

#### Perceived Educational Benefits

Paweł highlighted interactive activities such as rotating molecules, measuring angles, assessing polarity, and collaborative problem solving. Ana particularly emphasized the importance of improved spatial visualization, arguing that 3D models made molecular structures easier to understand and facilitated a later transition to 2D representations.

#### Technical Challenges

Paweł: The main technical challenges included the time-consuming process of setting up and logging multiple students into VR, difficulties with headset calibration and display sharpness, the need to reorganize the virtual classroom after breaks, and occasional Internet lag when several headsets were connected simultaneously or when large molecular models were used.

Ana: The main technical challenges involved headset disconnections, battery limitations, delays in loading and synchronizing virtual objects across users, students not always seeing molecular models immediately, and difficulties locating molecules that appeared in unexpected positions within the virtual environment.

#### Motion Sickness and Physical Comfort

Paweł reported only one student case of motion sickness and personally experienced substantial physical fatigue, eye swelling, and discomfort from the headset’s heat. Ana personally experienced severe dizziness during early VR use and in movement-based games. She found educational VR relatively comfortable and felt no more tired than after a traditional lesson, although many students became tired and complained about the headset’s weight.

#### Teacher Workload and Fatigue

Paweł found VR teaching significantly more exhausting than traditional teaching and felt unable to comfortably teach two VR classes in a single day. Ana considered VR teaching only slightly stressful initially and not particularly demanding once she became familiar with the system.

#### Student Reactions

Paweł described students as initially excited but also anxious and uncertain due to unfamiliarity with VR. Ana observed differences by age group: Primary school students were highly enthusiastic and curious. High school students were more reserved and sometimes ironic about the experience.

#### View on Motivation

Paweł did not explicitly discuss whether VR increased students’ motivation to study chemistry. Ana was skeptical that VR alone motivates students to study chemistry; she believed students were excited mainly because the experience was novel and exclusive rather than because it increased their interest in the subject itself.

#### Future Outlook

Paweł expressed interest in trying fully remote VR teaching in the future. Ana suggested improving VR environments by making them resemble real classrooms more closely to reduce the feeling of unfamiliarity and distraction.

### Students’ Opinions about Learning in VR

After the lesson, students completed a short questionnaire to express their attitudes toward working and learning in VR. Results are visible in Table 2. As we can see, students’ attitudes are rather positive. Students claim that they enjoy learning chemistry with VR. Additionally, they could better visualize molecular shapes.

**Table 2:** Mean and Median for Particular Questions^a^

This method is easy to work with.	3.92	4
I felt comfortable working with this method.	3.78	4
I enjoy learning chemistry this way.	4.31	5
I would like this method to be used more often in chemistry classes.	4.23	5
The classes helped me better understand the structure of molecules.	4.54	5
Thanks to the classes, I can better visualize the shape of molecules.	4.67	5
Thanks to the classes, it is easier for me to relate the properties of substances to their internal structure.	3.98	4
I would like this method to be used in other subjects as well.	4.10	5
Because I could move the molecules, I was more engaged in the class.	4.23	5
Because I could move the molecules, I was able to better understand the topics discussed.	4.33	4
This method is only suitable for fun/entertainment, not for learning chemistry.	2.01	2

a 1 – I definitely do not agree, 2 – I rather do not agree, 3 – It’s hard to say, 4 – I rather agree, 5 – I definitely agree.

### Still Trying to Have Fun

Although this lesson aimed to teach students about molecules, structure, and substance properties, we still wanted them to enjoy it and have fun. As shown in Figure 5, the self-ie moment is captured both in VR and in a real classroom, where students were taking a self-ie with the teacher. Additionally, several funny situations are described in Supporting Information 3.

**Figure 5:**
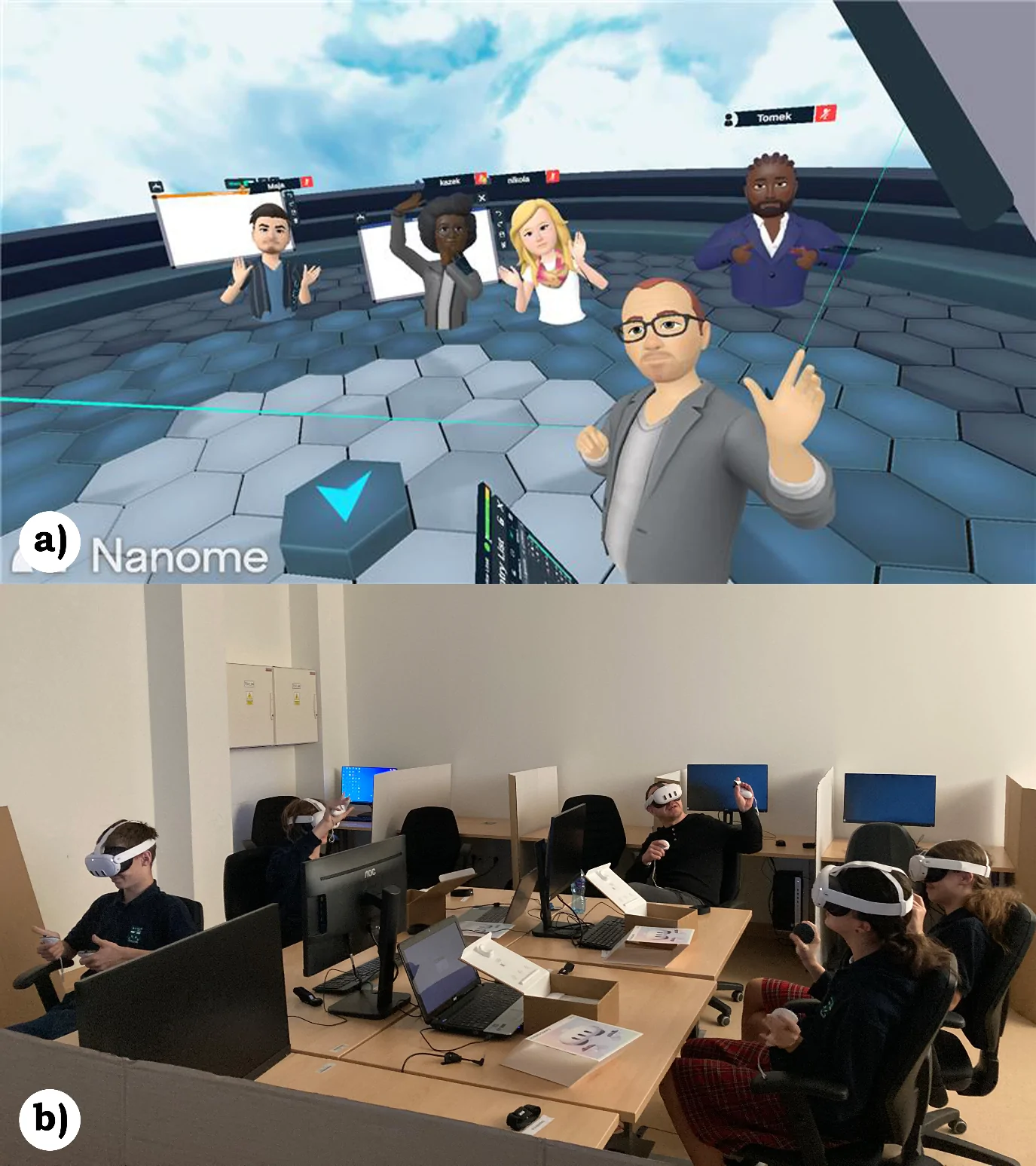
Making a self-ie in VR - the exact same moment: a) Taking a self-ie photo in VR; b) Taking a photo in real life.

## Discussion

While teaching and learning in VR are growing popular, successful implementation requires clear goals that address shared pedagogical challenges, whether the aim is motivation, concept visualization, or direct instruction. In secondary school chemistry, VR can solve equipment and chemical-access issues through virtual laboratories while uniquely elevating complex structural modeling. VR is often used in higher-level chemistry education, where students encounter a variety of experiences. Šmejkal et al. report similar challenges to implementing VR in preservice chemistry teacher training.^46^ They mention issues with the Internet connection and neck pain. However, students consider the VR environment engaging.

Similar results were reported in the Caribbean, where first-year undergraduate chemistry students used the MEL VR Chemistry app with a smartphone in a VR Headset. The authors demonstrated that VR can enhance students’ performance and engagement in chemistry.^47^ Surprisingly, they report no problems while using VR.

Yang et al. analyzed experience from implementing VR with junior high school students. They reported no challenges and confirmed that spherical video-based VR improved students’ learning achievement and increased their learning motivation, learning attitude, and creative thinking tendency.^48^

Garduño et al. present results on motivation to use VR in chemistry education in a high school science course, where students showed positive attitudes toward the motivation and acceptance of using virtual reality.^49^ Again, they report no particular problems with implementation.

Araiza-Alba et al. present a brief review of research published in 2019–2021 that considers the opportunities of VR in K-12 education.^50^ They conclude that research at this level of education is still lacking, since each learning group has its own specificities.

## Conclusions

Avoiding technology in education is not possible, and the best approach is to embrace it thoughtfully. This paper presents experiences teaching VR in secondary schools, along with insights from teachers’ perspectives.

Using VR in secondary school chemistry teaching is both promising and challenging. Schools often struggle with insufficient classroom equipment, difficulties with accessing and storing chemicals, etc. Virtual laboratories could help solve those problems. But we can go beyond laboratory issues. Virtual reality enables exceptional chemical-structure modeling. We can create large molecular models in seconds, play with them, modify them, calculate their properties, and literally step into them. That is something new and exciting. We should keep in mind that the software used in this study is not a typical educational environment. It is more of a research environment with an area for group collaboration. However, in our case, it worked very well, and the environment seems promising. Of course, in our scenario, many advanced features were not used because they are too complex for secondary school students. Still, our experience shows a clear market need for VR educational software focused on chemical models at this educational level.

Based on the experience, key challenges include equipment support, Internet connectivity, battery life, delays and synchronization problems, and device overheating. A crucial element is providing personal and technical support for the teacher. Additionally, the teacher must prepare the physical space, provide teaching and learning materials for themselves and the students, and allocate the required time. Interestingly, the process of drawing in air proved problematic. People often worry about virtual reality sickness, but we experienced it in our research only in a few cases. However, we must not overlook it. Students’ novelty response mattered: first-time VR users were more distracted by curiosity about the environment and controls; prior experience reduced this distraction. Overall, students’ opinions about VR chemistry learning are positive, and they see its potential in educational settings.

When we consider using VR in our classes, we must think about our goal. Is it to motivate students? Is it to teach them something? Or to help them visualize difficult elements? These are important questions we must consider before using VR because it requires a lot of time and patience. From the teachers’ perspective, VR is worth the effort once the method and hardware/software are mastered, because it becomes easier to reuse the environment and build additional lessons.

## Limitations

The research has a case study character and involves a limited number of participants (91), mostly from urban areas. A larger, more diverse sample would provide a broader perspective. Similarly, the teachers’ perspective is subjective, based only on two cases skilled in the educational use of ICT. The software used, Nanome, is research-focused, not specifically designed for educational implementation with secondary school students. Additionally, the topic taught in virtual reality (VR) offered limited opportunities to fully showcase VR’s benefits. Furthermore, this study does not cover analysis of learning outcomes.

**Ethical Statement**. Research received approval from the Rector’s Committee for Research Ethics of Jagiellonian University in Kraków under the number 1027.0041.4.2024.

## Supplementary Material



## Notes

Questions from the students’ questionnaire, detailed teachers’ perspectives, as well as funny situations that occurred during the lesson (PDF)
