# Supplementary material for: Teacher and Student Experiences during First-Trial Chemistry Lessons in Immersive Virtual Reality
Source: J Chem Educ. 2026 Oct 12;103(11):6672–81. doi: 10.1021/acs.jchemed.6c01221 (PMC13758927; doi:10.1021/acs.jchemed.6c01221)
Supplement: Supplementary file 1 [file ed6c01221_si_001.pdf]

Teacher and Student Experiences during First-Trial Chemistry Lessons in Immersive Virtual Reality

Mária Babinčáková,* Paweł Bernard

Jagiellonian University in Kraków, Faculty of Chemistry, Department of Chemical Education, ul. Gronostajowa 2, 30-387 Kraków, Poland

Supporting information 1: Questionnaire

After the session, students filled out a short, 5-point Likert scale questionnaire (1 – I definitely DO NOT agree, 2 – I rather DO NOT agree, 3 – It's hard to say, 4 – I rather agree, 5 – I definitely agree) to express their attitude towards learning in VR.

1. This method is easy to work with.
2. I felt comfortable working with this method.
3. I enjoy learning chemistry this way.
4. I would like this method to be used more often in chemistry classes.
5. The classes helped me better understand the structure of molecules.
6. Thanks to the classes, I can better visualize the shape of molecules.
7. Thanks to the classes, it is easier for me to relate the properties of substances to their internal structure.
8. I would like this method to be used in other subjects as well.
9. Because I could move the molecules, I was more engaged in the class.
10. Because I could move the molecules, I was able to better understand the topics discussed.
11. This method is only suitable for fun/entertainment, not for learning chemistry.
12. You can enter comments/additional information here: ………………………………

Supporting information 2: Teachers' experiences

Pawel's Experience

In general, I am enthusiastic about modern educational technologies and usually handle them quite well. Before using VR goggles for educational purposes, I had a chance to play with a VR set for half a year. Still, I recognize the transition of teaching into VR as quite

challenging. First of all, sets are designed for individual use. Without a proper management system, it is difficult to guide a group of students on how to open the system and log into the dedicated application. Unfortunately, it's not possible to prepare sets for immediate use in the app before the lesson. After putting on the googles, students must calibrate their position in the room and receive instructions on basic virtual reality operations. It should be noted that for most students, it was their first experience with VR, so they did not know how to operate the system and, at the beginning, experienced strong, mixed emotions. Most of them were very excited but also felt anxiety and uncertainty, not knowing what to expect. Therefore, getting into the app was the first challenge, even before teaching. On the other hand, once we get into the app, student management in Nanome is user-friendly and intuitive. We can arrange the group in a circle, with a central area dedicated to the virtual molecules we will work with. As a teacher, I am used to working with a blackboard and supporting my hand drawings with a presentation. In that manner, I arranged my virtual teaching area. Instead of an overhead projector, I used a web browser to open a personal website and upload a PDF presentation. In there, I had all the necessary data and exercises for students. Moreover, in the second tab, I was able to open the interactive periodic table (ptable.com) and use it similarly to classical classes. After the first use, it became clear that the classical teachers' blackboard was insufficient, so each student was asked to open and share their own board. In that way, I could provide information to the entire group, and each student could take their own notes and complete tasks independently. Moving virtually between students, I could check their notes and provide instant feedback. Since we were in the same classroom, we did not need microphones to communicate. However, I can imagine that each of us was somewhere else, running those classes remotely. Our discussions and task-solving were supported by virtual molecules whose structures and properties we analyzed. The Nanome environment allows the creation of a repository of molecules necessary for classes. I prefer to draw molecules in ChemSketch and send them to Nanome as SMILES codes rather than drawing them in Nanome; however, it is also possible. During the classes, I just pick the molecule from the repository created earlier and make it visible to everyone. During classes, students can take the model “in hand”, rotate it, enlarge it, measure angles, and make the basic calculations, e.g., polarity. Among many tasks,

students had to decide whether the chosen molecules are polar, draw them on boards using the wedge-and-dash representation, and design molecular structures with the given macroscopic properties of the substance. In most cases, sessions went very well. We experienced only one case of motion sickness, but in this case, the assistance of a second tutor in the classroom was necessary. However, the system has some limitations. First of all, students complained about the lack of sharpness in the presentation and the periodic table. Here, we deal with a problem of limited resolution in the app, as well as improper adjustment of the goggles. Users should adjust the distance between the lenses to match the distance between their pupils. Still, even with the verbal instruction provided at the beginning, some students had difficulty doing so or simply skipped it. Another problem is taking breaks during classes and removing headsets. After that, it's necessary to arrange the group in the virtual space again, and sometimes even log back into the app. It takes time and deconcentrates students. The final problem is the internet connection. Using 6 sets of goggles simultaneously can sometimes cause lag, especially when working with large molecules. As a teacher, after classes lasting 90 – 135 minutes, I felt quite tired, much more than after classical teaching. The heat generated by googles is pleasant at first, but over time it causes tiredness and swelling around the eyes. I was able to run classes day after day, but running two classes in a single day was too much for me. In summary, taking part in this study was very interesting and enriching. I would like to try to run a similar class remotely.

Ana's Experience

The second experience is from Ana, our colleague, who agreed to help us teach in VR. Ana had no previous experience in VR. With her, a semi-structured interview was conducted using the following question: Did you have a chance to work with VR before? How did you like it? How long did it take you to prepare for this one lesson? Do you think it was worth it? Was it demanding? What was the most difficult? What did you like? Do you think that it helped students? Do you think it motivates them? Here, key ideas are presented.

Ana had never worked with virtual reality before these lessons, not even recreationally. Her first real contact with VR came only after receiving the goggles from us, which allowed

her to test the technology before the lessons. She explored some of the games available on the device, but quickly discovered that adventure games caused severe dizziness and balance issues. Only simple games involving basic object manipulation felt comfortable for her.

Working in educational VR environments proved much more manageable, as long as she didn’t have to move extensively within the virtual space. She felt most comfortable when VR functioned like augmented reality (an extension of her real surroundings rather than a fully separate world). She particularly enjoyed the virtual classroom designed for students and the ability to manipulate molecular models.

Preparing for the lesson took her two longer sessions—roughly as long as the goggles' battery life, about two hours each. However, she didn’t prepare continuously; instead, she often entered the VR environment for short periods, reflected on her ideas, and then returned to verify different didactic solutions. She spent more time fine-tuning the teaching concept than learning how to use the software. One difficulty she encountered was that the molecules often appeared in unpredictable locations within the VR space, requiring practice to find them quickly.

During the lessons, the biggest challenges were technical: the goggles sometimes disconnected, ran out of battery, or displayed objects with noticeable delays. Students also did not always see the molecular model immediately, even when it was already visible to her. Additionally, some students became distracted, exploring menus or experimenting with unrelated features instead of following the lesson.

Despite these issues, according to Ana, VR offered clear advantages. The 3D molecular models were much easier to understand than those shown on the board or in textbooks, which made the transition to 2D drawings simpler afterward. Students could easily visualize the true spatial structure, something that is usually difficult to convey in traditional lessons.

Interestingly, she conducted VR lessons with both primary and high school students. In her view, primary school children were far more enthusiastic and curious, while high school students often approached VR with a distance—or even mild irony.

From her own perspective, teaching with VR was not particularly exhausting. The most uncomfortable aspect was having to remain seated. She felt similarly tired afterward as she would after a traditional class. Students, however, did become tired toward the end and sometimes complained about the weight of the goggles. During the first lessons, she felt stressed due to the technical uncertainty, but this quickly faded as she gained confidence.

She believes that the time invested in preparing to work with VR was worthwhile, since once the teacher learns the system, new lessons can be created more efficiently. She wondered, however, to what extent the “foreignness” of the virtual environment helps or distracts students. She suggested that if the VR space could visually resemble their real classroom—while still displaying 3D models—it might feel more natural for learners.

When asked whether VR could motivate students to study chemistry, she said that students did not suddenly express interest in studying chemistry solely because of the VR sessions, but they did feel special for being selected to participate and were enthusiastic about the experience. Some were more excited about owning such goggles for entertainment than for learning.

Supporting information 3: Funny Situations from teaching

During the classes, many funny situations appeared:

- entering VR for the first time:

Students: *"Oooooo, that's cool! And weird!"*

- Not seeing VR space as a one where we can move:

Teacher: *"I would like to ask you to move a little bit further."*

The student gets up from the chair.

Teacher: *"No! In VR."*

Teacher: *"Maria, move closer to Jagoda."*

Student gets up and grabs the chair: *"Should I also take the whiteboard with me?"*

Teacher: *"Not in reality!"*

Student: *"Asiu, please get away from me. You are very close."*

Student 2: *"But it is in VR, not in real. Why do you mind?"*

- When normal situations appear as something special:

Teacher: *"And now we will have a whiteboard in front of us."*

Students: *"Wooow!"*

- When it is difficult to work in VR

Student1: *"Ah, that's ugly!"*

Teacher: *"Did you finish? Can you show me?"*

Student 2: *"Yes, but you will definitely get scared when you see it."*

- Sometimes the molecules appear somewhere in the class, not in front of you.

Teacher: *"And now everybody is searching for our molecule. Guys, we don't have a molecule. We must find it. Ah, ok, let it be."*

Student: "*There it is! Above you!"*

- When you have goggles on the whole lesson, and you don't know what your students look like.

Student after the lesson: *"I need some help."*

Teacher: *"Which one are you?"*

Students: *"Eryk"*

Teacher: *"But which one are you? I don't know how you look. I recognize only your avatar."*

Learning Objectives

- To practice the skill of relating the structure of matter to its properties. (How does the structure of a molecule—whether polar or nonpolar—affect the physical properties of a substance?)
- To review the concept of chemical bond polarity. To familiarize students with the method for determining the polarity of chemical molecules.
- To familiarize students with the notation of structural formulas that take into account the three-dimensional structure of a molecule (bonds in front of and behind the "plane of the page").
- To familiarize students with the concepts of optical isomerism, chirality, enantiomers, and the absolute configuration of R and S isomers; and the notation of structural formulas that take into account the three-dimensional structure of a molecule (bonds in front of and behind the "plane of the page").
- Practicing the skill of mentally manipulating models of chemical molecules.

Operational Objectives

After the lesson, the student will be able to:

- Describe the electron configuration/ structure of the molecules H_2O, CH_4, CH_3F, CH_2F_2, CHF_3, CF_4, and CH_3OH; describe their spatial structure; determine the polarity of the molecules; and relate it to the properties of the substances—in particular, their state of matter and boiling point.
- Build models of the above molecules using VR headset app.
- Draw molecular formulas that reflect their three-dimensional structure, using bold and dashed lines to indicate atoms that are not in the plane of the paper.
- Name enantiomers based on their absolute configuration.
- Solve problems that require creating a three-dimensional mental model of simple molecules and manipulating that model in one's imagination.

Molecular Polarity

Water

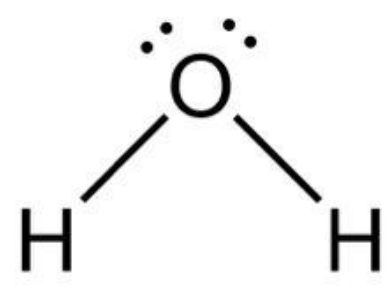

1. What is the molecular formula for water? What atoms is it made of? How many protons and how many electrons do they have?
2. Draw the electron dot structure of water and describe how the bonds are formed.
3. Build a 3D model of the molecule using VR goggles.
4. Based on the electronegativity values, identify the type of bonds present in a water molecule.
5. How are the charges distributed in the bonds?
6. What is the shape of the molecule?

7. Is a water molecule polar?
8. Where is the positive charge located, and where is the negative charge located in the molecule?
9. Why do we describe water molecules as dipoles? (Explain the polarity of a water molecule in terms of the distribution of charges in the bonds and the molecule's geometry.)
10. Is molecular polarity important? How does molecular polarity affect the physical properties of water? (Refer to the boiling point of water and the role of water as a solvent.)
11. The boiling and melting points of water compared to other molecules with similar mass but lower polarity (example: H_2S, CH_4. For H_2S, note that the molecular structure is analogous to that of water; for CH_4, simply compare bond polarity and boiling points without analyzing the molecular structure; for H_2S, $\Delta E = 0.38$, and for CH_4, $\Delta E = 0.35$.)

	Boiling point	Solubility in water
H_2S	−59.55 °C	3.980 g/l (20 °C)
CH_4	−161.48 °C	0.022 g/l (17 °C)

CH_4

1. Let's continue discussing the properties of CH_4. $\Delta E = 0.38$ for H_2S, and for CH_4, $\Delta E = 0.35$—so why is the difference in boiling points over 100 °C? To answer this question, we need to consider the structure of this molecule.
2. What atoms is it made of? How many protons and how many electrons do they have?
3. Draw the structural formula of the molecule and describe how the bonds are formed.
4. Build a 3D model of the molecule using VR goggles.
5. Based on the electronegativity difference, identify the type of bond.
6. What is the shape of the molecule?
7. How are the charges distributed in the bonds?
8. Where is the positive charge located, and where is the negative charge located in the molecule?
9. Is the molecule polar? (Determine the molecule's polarity based on the charge distribution in the bonds and the molecule's geometry.)
10. How does the structure of this molecule affect the boiling point and solubility of this substance in water?

Representing 3D molecular structures in 2D

We have examined the structure of selected chemical molecules and analyzed how their structure affects their properties. Let's now consider how to represent a molecule's structure on a piece of paper so that the notation conveys its three-dimensional structure.

Let's return to the example of methane—CH_4

Do you know its molecular formula? (Let's draw the formulas:)

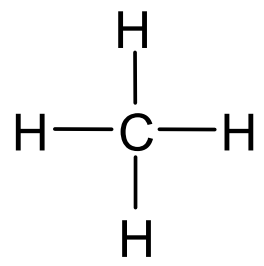

And the structure of the molecule. This molecule has the shape of a tetrahedron with a carbon atom at its center.

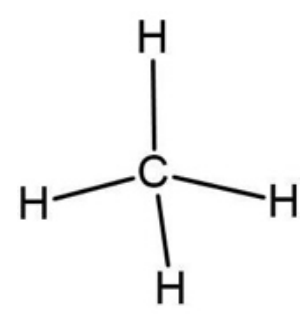

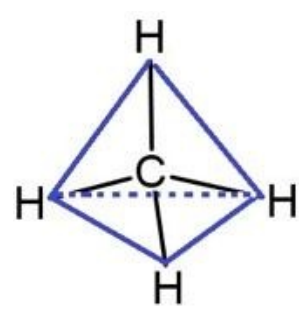

The blue lines are only meant to help you visualize the shape of the tetrahedron.

To represent the three-dimensional arrangement of the molecule on a flat sheet of paper, we use different types of lines to indicate the orientation of the bonds.

A straight bond lies in the plane of the paper.	A wedge, or a bold line, indicates that the bond "extends out of the page" toward our eyes (it is in front of the plane of the page).	A dashed line indicates that the bond "passes through the paper" and moves away from us (it is behind the plane of the paper).

We position the methane molecule model accordingly and draw the formula:

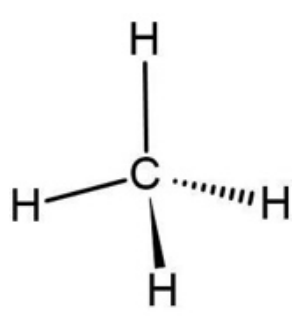

CH_3F, CH_2F_2, CHF_3, CF_4 , CH_3OH combined with the three-dimensional structure (next point)

1. How will the properties of methane change if we replace one of the hydrogen atoms with a fluorine atom? – A question for students without an explanation at this stage. Verification of statements based on an analysis of the substance's structure in the following steps.
2. What atoms is it made of? How many valence electrons does fluorine have? What is the electronegativity of fluorine compared to that of H and C atoms?
3. Draw the structural formula of the molecule and describe how the bonds are formed.
4. Build a 3D model of the molecule using or VR goggles.
5. Based on the electronegativity difference, identify the bond type.
6. What is the shape of the molecule?
7. How are the charges distributed in the bonds?
8. Where is the positive charge located, and where is the negative charge located in the molecule?
9. Is the molecule polar? (Determine the polarity of molecules based on the distribution of charges in the bonds and the molecule's geometry.)
10. How does the structure of this molecule affect the boiling point and water solubility of this substance?
11. We repeat the analysis for the molecules CH_2F_2, CHF_3, and CF_4 (items 3–10)

	Dipole moment	Boiling point	Solubility in water
**CH_4**	0 D	-161.48 °C	0.022 g/L
**CH_3 F**	1.8 D	-78.4 °C	2.295 g/L
**CH_2F_2**	1.9 D	-51.6 °C	3.628 g/L
**CHF_3**	1.65 D	-82.1 °C	1.000 g/l
**CF_4**	0 D	-127.8 °C	0.020 g/l

Exercise: CH_3F model

1. Build a model of the CH_3 F molecule in VR
2. Place the model in various positions and <u>practice writing 2D formulas. Rotate it around the bond axis.</u>

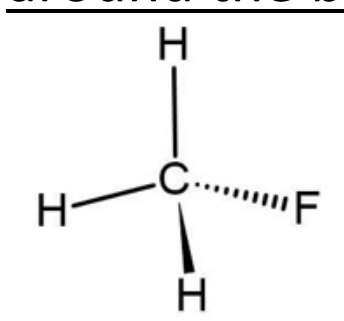
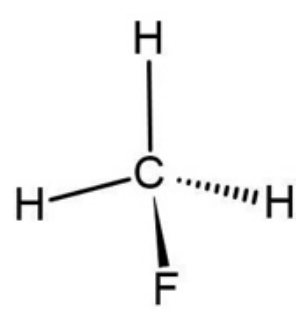
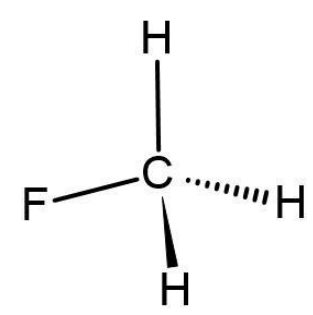
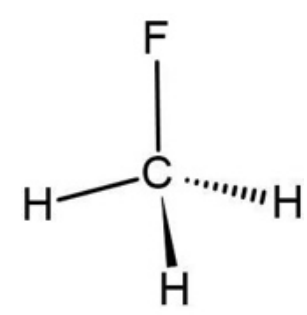

3. Do the drawings represent the same molecule?

Exercise: CH_2BrF model

1. Build a model of the CH_2BrF molecule in VR
2. We position the model in different orientations and practice writing 2D structural formulas.

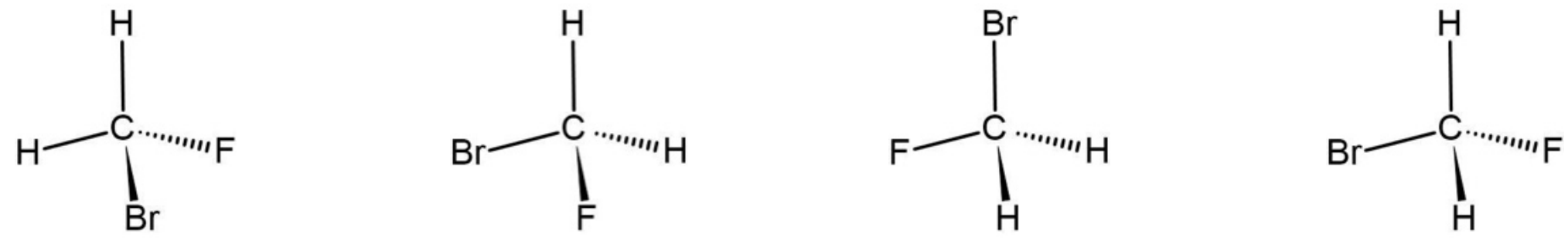

3. Do the drawings represent the same molecule?

Writing spatial formulas based on models

1. We position the models in the given orientations, and students draw the structures

**A** 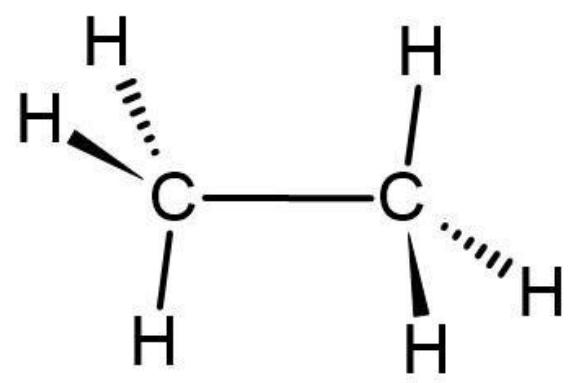	**B** 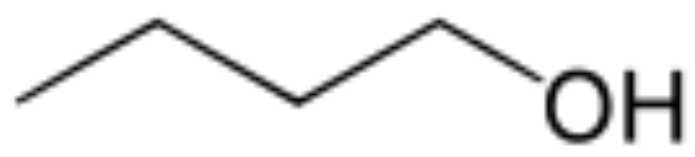	**C** 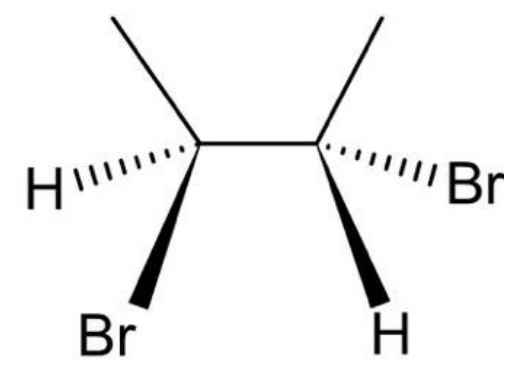

Enantiomers – the R and S system

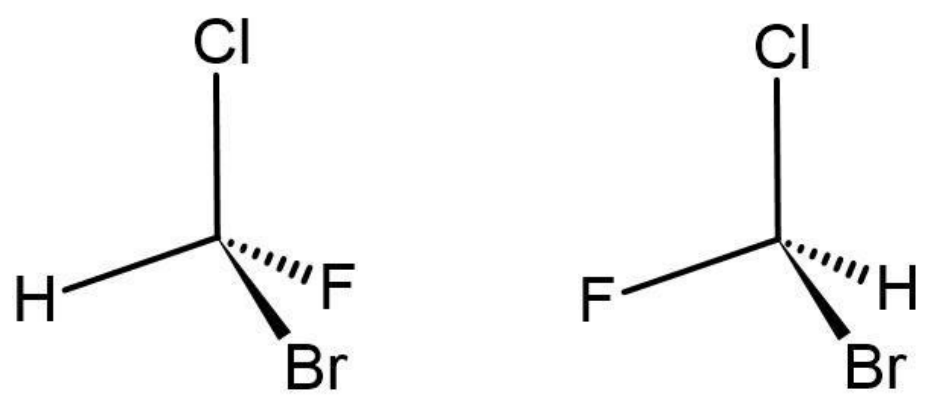

1. What is the name of the molecule in the figure?
2. Are they the same?
3. Build the molecule shown in the figure.
4. Can the molecules be superimposed on each other? Is this the same molecule, or are they isomers?
5. We introduce the concepts of a chiral carbon atom and a chiral molecule.
6. We introduce the concept of enantiomers—a pair of non-superimposable chemical molecules that are mirror images of each other (similar to the right and left hands).
7. We must therefore name these two isomers so that we know which one we are referring to.
8. We introduce a naming system based on absolute configuration and the CIP convention.

Exercise: CBrClFI model

1. Write, draw, and construct both enantiomeric forms of bromochlorofluoroiodomethane

Exercise:

1. Consider the given pair of structures and determine whether they are enantiomers or the same molecule that has simply been rotated in space. Assign the correct configuration (R) or (S) to each of them.

	Structure 1	Structure 1	Conclusion
**A.**	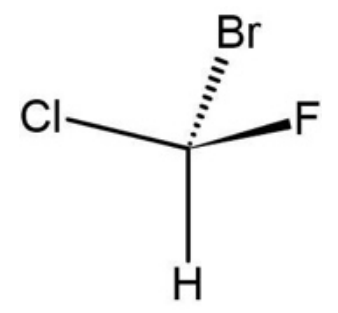	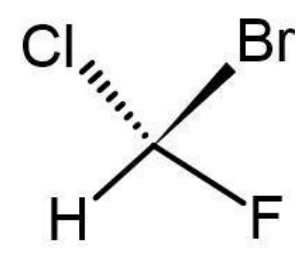	
**B.**	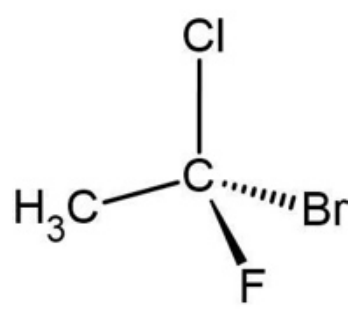	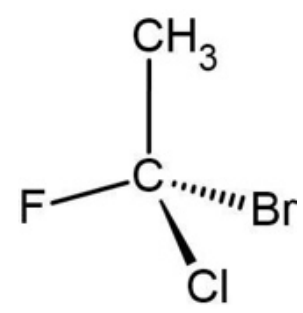	
**C.**	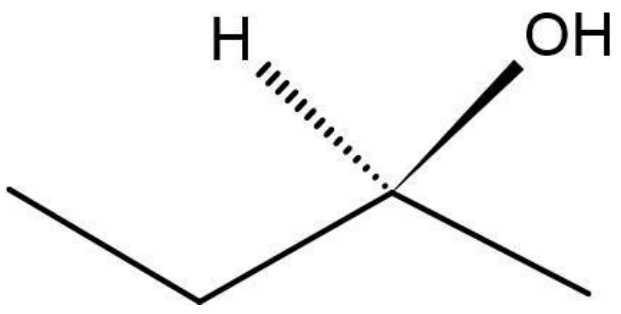	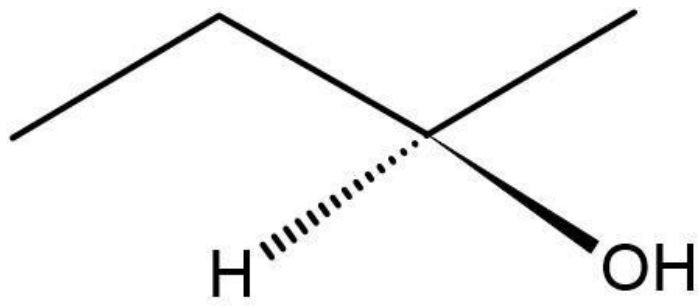	

Rotation and Docking Exercises:

1. Bioactivity of enantiomers

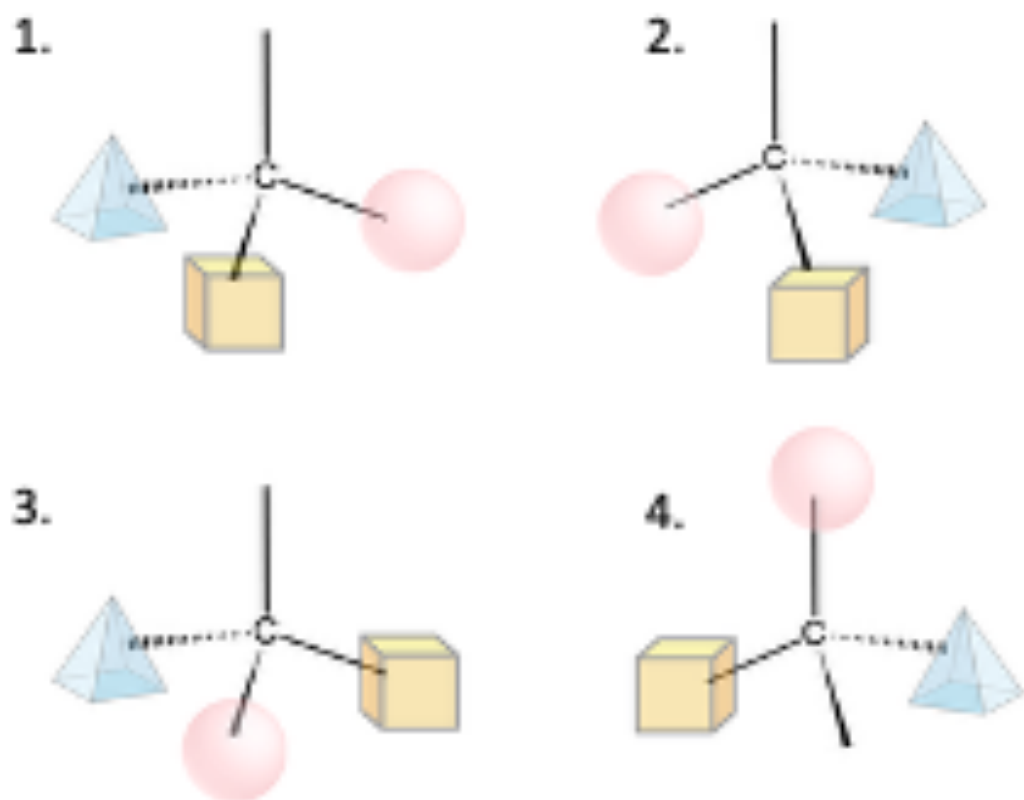

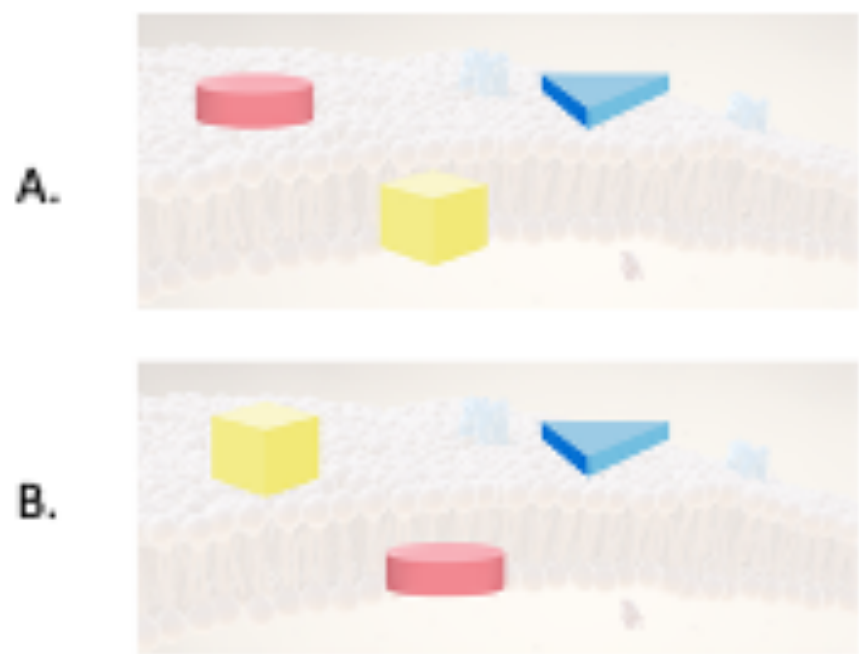

Review

1. Is a molecule always polar when it contains polar covalent bonds? Explain why.
2. How does the polarity of molecules affect a substance's solubility in water?
3. What factors determine a substance's boiling point?
4. What does a chemical bond represented by a wedge in a structural formula mean? What does a dashed line represent, and what does a solid line represent?
